# Vacuum Brazing of C/C Composite and TiAl Intermetallic Alloy Using BNi-2 Brazing Filler Metal

**DOI:** 10.3390/ma14081844

**Published:** 2021-04-08

**Authors:** Shengnan Li, Dong Du, Lei Zhang, Qingle Hao, Weimin Long

**Affiliations:** 1 Department of Mechanical Engineering, Tsinghua University, Beijing 100084, China; lisn16@mails.tsinghua.edu.cn (S.L.); dudong@tsinghua.edu.cn (D.D.); zhanglei86529029@sina.com (L.Z.); 2 State Key Laboratory of Advanced Brazing Filler Metals & Technology, Zhengzhou Research Institute of Mechanical Engineering Co., LTD, Zhengzhou 450001, China; hao_ql@126.com; 3 China Innovation Academy of Intelligent Equipment (Ningbo) Co., LTD, Ningbo 315700, China

**Keywords:** carbon/carbon composite, TiAl intermetallic compound, BNi-2 brazing filler metal, high-temperature vacuum brazing

## Abstract

C/C composite was brazed to TiAl intermetallic compound using a commercial BNi-2 brazing filler metal under vacuum brazing condition. The brazing temperature was 1030~1150 °C and the holding time was 20 min. The joint interfacial microstructures and mechanical properties were studied, and the fracture behavior and joining mechanism were also investigated. The effect of brazing temperature on the joint shear strength was explored. The results showed that a perfect interface joint can be obtained by using BNi-2 to braze C/C and TiAl. During brazing, Ti, Cr, and other carbide forming elements diffused to C/C composite side, forming Cr_3_C_2_, Cr_7_C_3_, TiC, and other carbides, and realizing metallurgical joining between the brazing filler metal and C/C composite. The microstructure of the interface of C/C composite and TiAl intermetallic compound joint is as follows: TiAl alloy → TiAl + AlNi_3_ → AlNi_2_Ti → Ni(s, s) + Ti_3_Al + Ni_3_Si → Ni(s, s) + Ni_3_(Si, B) + CrB → Ni(s, s) + Ni_3_Si + TiCr_2_ → (Ti, Cr)C → C/C composite. When the holding time is fixed, with the increase of brazing temperature, the shear strength of the joint increases first and then decreases. The maximum average room temperature shear strength of the brazed joint was 11.62 MPa, while the brazing temperature was 1060 °C and the holding time was 20 min.

## 1. Introduction

It is difficult and expensive to directly prepare large and complex C/C components because of the complex preparation process and long generation cycle of C/C composites. Meanwhile, the material itself has poor plasticity, difficult deformation and poor workability. The individual use of C/C composite is limited to a certain extent. Generally, the economical and viable solution is joining C/C composite to metals or other materials [1,2]. Ti-Al series intermetallic compound (IMC) is one kind of new high-temperature structural material developed in recent years. It possesses the advantages of low density, high specific strength and stiffness, excellent high temperature performance, strong creep resistance and oxidation resistance. It is a new high-temperature material which has the potential to replace Ti-based alloy and Ni-based superalloy in aviation and aerospace [3,4]. Joining C/C composite and TiAl IMC together can achieve satisfactory high-temperature performance, greatly reduce components’ weight and energy consumption, because of these two materials have complementary advantages. It is of great significance to intensive study the bonding of these two materials and optimize the joint performance.

Wang et al. [5] brazed C/C composite and TiAl alloy using Ag-26.7Cu-4.6Ti (wt.%) brazing filler metal under vacuum condition. The highest shear strength of the joint obtained was 12.9 MPa under 900 °C/10 min brazing specification. The shear strength of the joint was increased to 26.4 MPa after surface drilling treatment of C/C composite. Cao et al. [6] brazed C/C composite with TiAl alloy using (Ti/Ni/Cu)_f_ multi-layer foil filler and achieved a good interface integration. Under the brazing specification of 980 °C/10 min, the room temperature shear strength of C/C-TiAl reached a maximum of 18 MPa, and the 600 °C high-temperature shear strength of the joint was 22 MPa.

Cao et al. [7] prepared TiAl alloy using high-temperature self-propagating combustion synthesis method and realized the joining between TiAl alloy and C/C composite simultaneously. The effect of stress on joint interface was studied through embedding C/C composite pellet into Ti-45Al (mol.%) mixed powder and filling TiAl powder mixture into a hollow C/C composite ball, respectively. When C/C composite pellet was put into Ti-Al mixed powder, after combustion and synthesis, the interface was successfully bonded due to residual compressive stress at the interface. On the contrary, when filling Ti-Al mixed powder into a hollow C/C composite ball, the interface was under the action of tensile stress, and the joint cracked along the interface. A kind of crack-free TiAl-C/C joint was obtained through experiments. The interfacial microstructure was composed of TiAl, TiAl_3_, TiC compounds and C/C composite.

Adopting high-temperature self-propagating combustion synthesis method, Cao et al. [8] also realized the joining between C/C composite and TiAl intermetallic compound while using Ti-Al-C powder and Ag-26.7Cu-4.6Ti (wt.%) filler foil as the intermediate layer materials. The influence of reactant composition and preheating temperature on adiabatic temperature was calculated. It is proposed that the heat generated by the reaction is determined by the composition of the intermediate layer ((m + 1)Ti-3Al-mC), m value). The highest shear strength of the joint was 17.6 MPa when the thickness between layers was 500 μm and m = 1.1. There was a TiC layer on C/C composite side and a Ti-Al-Cu layer on TiAl side respectively according to the analysis results of the joint microstructure composition.

Guo et al. [9] achieved vacuum brazing of C/C composite and Ti_3_Al alloy using graphene nanoplatelet (GNP) strengthened Ag-26.7Cu-4.6Ti (wt.%) brazing filler metal under 880 °C/10 min brazing specification. The brazed joint had excellent bonding interface, and the microstructure of joint was composed of TiC, Ti(Cu,Al), GNPs, AlCu_2_Ti phases. The addition of GNPs could decrease the thickness of TiC layer. GNPs hindered the atom diffusion and inhibited the growth of TiCu phase on Ti_3_Al alloy side. The shear strength of brazed joint first increased and then decreased with the increase of GNPs content, and the brazed joint reached to the highest shear strength 26.7 MPa when GNPs content was 0.1 wt.%. GNPs could also reduce the coefficient of thermal expansion (CTE) mismatch between C/C composite and Ti_3_Al alloy, and improve the joint strength due to the relief of residual stress.

In conclusion, as for the joining between C/C composite and Ti-Al IMC, the high-temperature self-propagating combustion synthesis method and brazing method are mainly adopted at present. While brazing these two materials, Ag-Cu-Ti alloys are the mostly used brazing filler metals. However, the working temperature of brazed joint prepared by Ag-based, Cu-based brazing filler metal is generally no more than 400 °C while joining ceramic and metal through directly active brazing. If the service temperature of brazed joint exceeds 400 °C (such as 400~800 °C), Au-based, Pd-based, or Ti-based filler metals should be selected. The joint brazed with filler metals based on Ni, Co high temperature metals can be qualified for 800~1000 °C working temperature [10]. Therefore, the brazed joint prepared by Ag-Cu-Ti filler metal has low service temperature, which cannot reflect the excellent high-temperature performance of the base materials. There are few related reports on the high-temperature brazing of these two materials. The service temperature of brazed joint that prepared by Ni-based brazing filler metal is higher, and Ni-based brazing filler metal is commonly used in aerospace field. In this paper, BNi-2 high-temperature brazing filler metal was used to braze C/C composite and TiAl alloy under vacuum atmosphere to explore an effective way to improve the service temperature of the joint.

## 2. Materials and Methods

**Figure 1:**
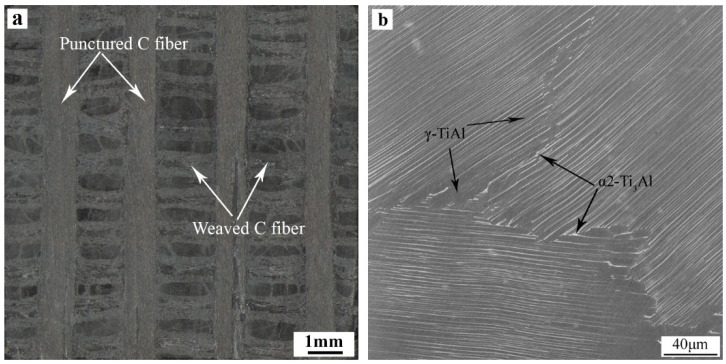
Morphologies of base materials: (**a**) Optical (OM) macro-morphology of C/C composite, (**b**) Backscattered electron (BSE) micromorphology of TiAl alloy.

The C/C composite used in this experiment is quasi-three-dimensional C/C composite. The carbon fiber prefabricated body is two-dimensionally (2-D) entwined and 3-D punctured, and the matrix is graphite obtained by impregnation cracking and graphitization. The density of C/C composite is about 1.8 g/cm^3^. The shear strength perpendicular to the puncture direction is about 35~40 MPa, and the interlaminar shear strength is about 15 MPa (Figure 1a). TiAl intermetallic (IMC) is Ti-47.5Al-2.5V-1Cr-0.2Zr (at.%) alloy. The microstructures are oriented lamellar tissues composed of near lamellar structure, including γ-TiAl and α2-Ti_3_Al dimorphic tissues. The volume fraction of γ grain is about 5% (Figure 1b). The basic properties of C/C composite and TiAl alloy are shown in Table 1.

C/C composite was sliced into 10 mm × 5 mm × 5 mm pieces. TiAl alloy was cut into 10 mm × 10 mm × 5 mm slices for the metallographic observation and 15 mm × 10 mm × 5 mm for shear test, respectively. The TiAl slices were polished by SiC papers up to grit 800 to remove surface debris. TiAl alloy and C/C composite samples were ultrasonic cleaned in alcohol for 15 min and dried before brazing. Commercial Ni-base braze powders (<48 μm) with a nominal composition of Ni-(6.0~8.0)Cr-(4.0~5.0)Si-(2.75~3.50)B-(2.5~3.5)Fe (wt.%) was used in this experiment. The filler metal powder was mixed into paste with trichloroethylene before using.

C/C composite, BNi-2 filler metal and TiAl alloy were assembled according to sandwich structure. The assembly was heated to 1030 °C, 1060 °C, 1090 °C, 1120 °C, and 1150 °C in a vacuum furnace and isothermally held for 20 min while the vacuum degree was (2~5) × 10^−3^ Pa and then cooled in furnace.

**Figure 2:**
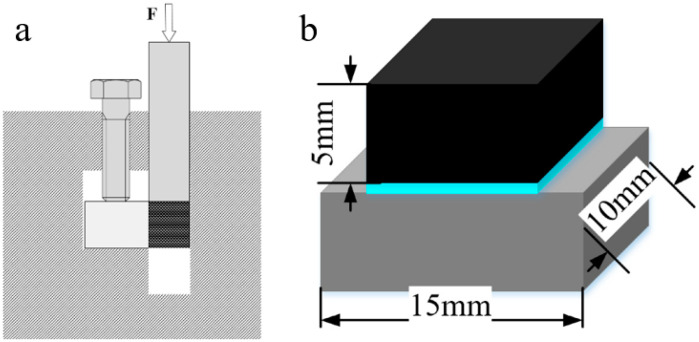
(**a**) Schematic of shear test setup (**b**) Geometry of shear sample of brazed joint.

The cross-sectional microstructure of brazed joint was characterized by Phenom (Phenom XL) desk scanning electron microscope (SEM) (Thermo Fisher Scientific, Waltham, MA, USA) coupled with energy disperse spectroscopy (EDS). The interfacial phase composition was analyzed using EDS and X-Ray diffraction (XRD). A Bruker D8 Advance XRD apparatus (XRD, Cu Kα) (Bruker, Billerica, MA, USA) was used for XRD testing experiments. The scanning angle range was 10~100° with a speed of 2°/min. The shear strength of the joints at room temperature was examined using an MTS E45.105 universal testing machine. During the test, the moving speed of the chuck was 0.1 mm/s. The maximum load at fracture was recorded and the shear strength of the joint was calculated according to formula (1). Schematic of the setup used for shear test and the specimen geometry is shown in Figure 2. Average strength was determined from five tests of shear samples that were achieved under the same conditions. After shear testing, the fracture surface of brazed joint was observed by SEM.

(1) $$\tau =\frac{F}{S}$$

*τ*: Shear strength of the brazed joint (MPa),
*F*: Fracture load measured in the shear test (kN),
*S*: Effective cross-sectional area of the joint surface (mm^2^).

## 3. Results and Discussions

### 3.1. Microstructure and Composition of C/C-BNi2-TiAl Brazed Joint

**Figure 3:**
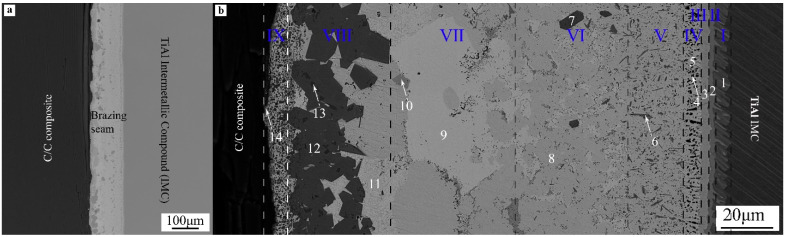
BSE Morphologies of C/C-BNi2-TiAl brazed joint under 1060 °C/20 min parameter: (**a**) Overall morphology of the joint, (**b**) Cross section morphology of the joint.

The overall morphology of the CC-BNi2-TiAl joint interface brazed under the brazing temperature of 1060 °C and the holding time of 20 min is shown in Figure 3. It can be seen from Figure 3a that vacuum brazing of C/C composite and TiAl alloy can be realized by using BNi-2 filler metal. The interface of brazed joint was well combined without obvious crack defects. Layered transition morphology was appeared in the internal interface of the joint from cross section morphology (Figure 3b). During brazing, active Ti element and Al element dissolved from the initial matrix entered the molten filler metal, resulting in chemical reactions between brazing filler metal and base material, and new reaction layers on the interface. The width of the whole joint was about 150 μm. From TiAl alloy to C/C composite side, there were roughly nine layers with different morphologies according to the differences in microstructure.

EDS analyses for different characteristic areas in Figure 3 were carried out, the results are shown in Table 2.

It can be inferred that Ti-Al, Ti-Ni, Al-Ni binary and Ti-Al-Ni ternary compounds were generated because of the chemical reactions on TiAl alloy interface according to EDS analyses results in Table 2. Reaction layer V~VII in Figure 3b, the main microstructure was Ni base solid solution Ni(s, s) (region 9), some small particulate phases were dispersive distributed on Ni(s, s) matrix. The microstructure of BNi-2 alloy is based on Ni-based solid solution Ni(s, s) as the matrix, and Ni_3_Si, Ni_3_B, CrB phased distributed on the surface of Ni(s, s) [13]. Combined with the microstructure morphology and EDS analyses results, the composition of reaction layers V~VII was almost the same with BNi-2 alloy. The enrichment of Ti and Cr elements occured at the interface of C/C composite side due to high chemical affinity between Ti, Cr elements and C elements. A reaction layer dominated by carbide (Ti, Cr)C was formed.

**Figure 4:**
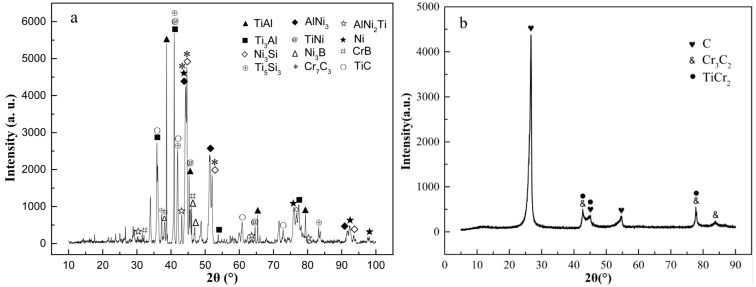
XRD test results of brazed joint under 1060 °C/20 min parameter: (**a**) TiAl side, (**b**) C/C composite side [14,15,16,17,18,19,20,21,22,23,24,25,26,27,28].

XRD detection and analyses of braze joint prepared under 1060 °C/20 min condition were carried out on both TiAl side and C/C composite side of the reaction interfaces for further determination of the type of generated compounds. The analysis results are shown in Figure 4.

XRD test results showed that the main phases on TiAl side of the brazed joint were composed of Ti_3_Al, TiAl, AlNi_3_, TiNi binary intermetallic compounds and AlNi_2_Ti ternary intermetallic compound. The phase composition of the brazing seam center was similar to that of BNi-2 filler metal, Ni_3_Si, Ni_3_B, CrB and other compounds are dispersed on Ni-based solid solution Ni(s, s). Element B was detected in the energy spectrum analysis results of microzone 6 and 10. However B is a light element, EDS analysis could not accurately determine its content, while XRD test results confirmed its existence. XRD test results also show that the intermetallic compounds such as Ti_5_Si_3_ and TiCr_2_ were formed by the reaction of the filler elements with Ti during brazing.

On C/C composite side, strong carbide forming elements were enriched and reacted with C to generate Cr_3_C_2_, Cr_7_C_3_, TiC, and other compounds (region 13, 14). These compounds were small particles and in a diffuse distribution state near C/C composite side (transition layer IX). The main phase of transition layer VIII was the shallow black area 12, which can be concluded as TiCr_2_ phase combining with EDS and XRD analysis results. The reaction between Ti and Cr elements in this transition layer inhibited the overreaction of these two elements with C, avoid the brittleness of the joint caused by excessive or continuous carbide content on C/C composite side.

The formation of carbides and the relationships between Gibbs free energies (ΔG) and temperatures are shown as Equations (2)–(4).

3Cr(s) + 2C(s) → Cr_3_C_2_(s), ΔG_Cr3C2_ = −94.140 − 0.003T (2)

7Cr(s) + 3C(s) → Cr_7_C_3_(s), ΔG_Cr7C3_ = −181.167 − 0.018T (3)

Ti(s) + C(s) → TiC(s), ΔG_TiC_ = −184.096 + 0.012T (4)

Thermodynamic calculation shows that the Gibbs free energy of the carbide generated in the above formulas at 1060 °C is: ΔG_Cr3C2_ = −98.14 kJ/mol, ΔG_Cr7C3_ = −205.16 kJ/mol, ΔG_TiC_ = −168.10 kJ/mol, respectively. Therefore, the thermodynamic possibility of the formation of these carbides during the reaction has been indicated.

In conclusion, the vacuum brazing between C/C composite and TiAl alloy can be realized by using BNi-2 filler metal at the brazing temperature of 1060 °C and the holding time of 20 min. Multi-layered transition morphology was appeared in the joint interface, and the interface was well combined without obvious inside crack defects. Through EDS analyses of specific areas and XRD analyses of the interfaces on both sides, during the brazing process, chemical reactions were occurred between different elements on the joint interface. Binary or ternary compounds were generated, and metallurgical joining of the joint interface was realized. The microstructure of the interface of C/C composite and TiAl intermetallic compound joint is as follows: TiAl alloy → TiAl + AlNi_3_ → AlNi_2_Ti → Ni(s, s) + Ti_3_Al + Ni_3_Si → Ni(s, s) + Ni_3_(Si, B) + CrB → Ni(s, s) + Ni_3_Si + TiCr_2_ → (Ti, Cr)C → C/C composite.

### 3.2. Influence of Brazing Temperature on Microstructure and Properties of Brazed Joint

#### 3.2.1. Influence of Brazing Temperature on Mechanical Properties of Brazed Joint

**Figure 5:**
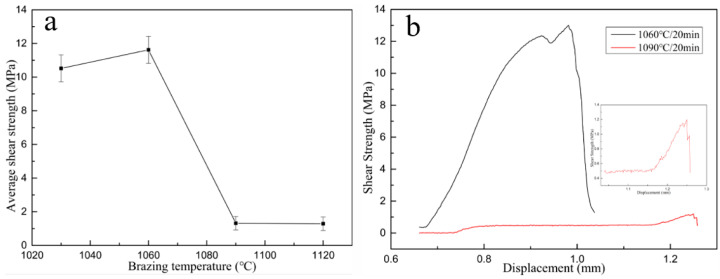
(**a**) Effect of brazing temperature on shear strength, (**b**) Shear strength curve of brazed joint.

Figure 5 shows the change of average shear strength of the joint according to the brazing temperature while keeping the same heating time. When the brazing temperature was 1150 °C, cracks were appeared in the brazed joint. The shear strength of brazed joint was almost zero, that is the brazed joint had no strength.

The room temperature shear strength of the joint first increased and then decreased as the brazing temperature increased under the same holding time period. When the brazing temperature was 1060 °C and holding time was 20 min, the average shear strength of the joint reached the highest, which was 11.62 MPa. When brazing temperature was higher than 1060 °C, the shear strength decreased sharply, which was greatly related to the amount, distribution pattern of interfacial compounds, and the residual stress of the joint.

The shear strength curves of the joint are shown in Figure 5b. The load decreased perpendicularly when the brazing parameter was 1090 °C/20 min, and the shear strength of brazed joint was extremely low. Brazed joint prepared under 1060 °C presented good toughness to some extent compared with prepared under 1090 °C. This may be caused by thickness of the reaction layer and the distribution of the carbides and will be explained in detail in the following paragraph.

#### 3.2.2. Influence of Brazing Temperature on Microstructure of Brazed Joint

**Figure 6:**
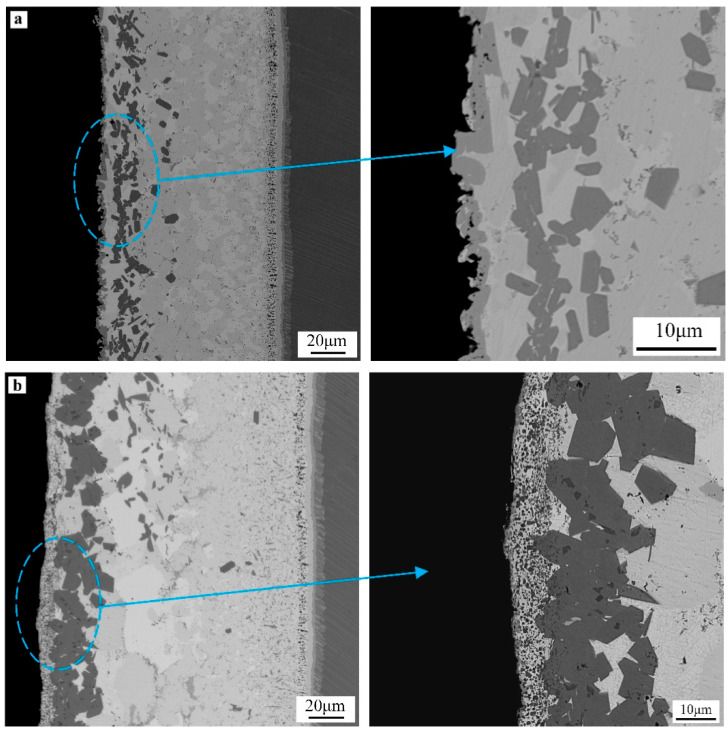

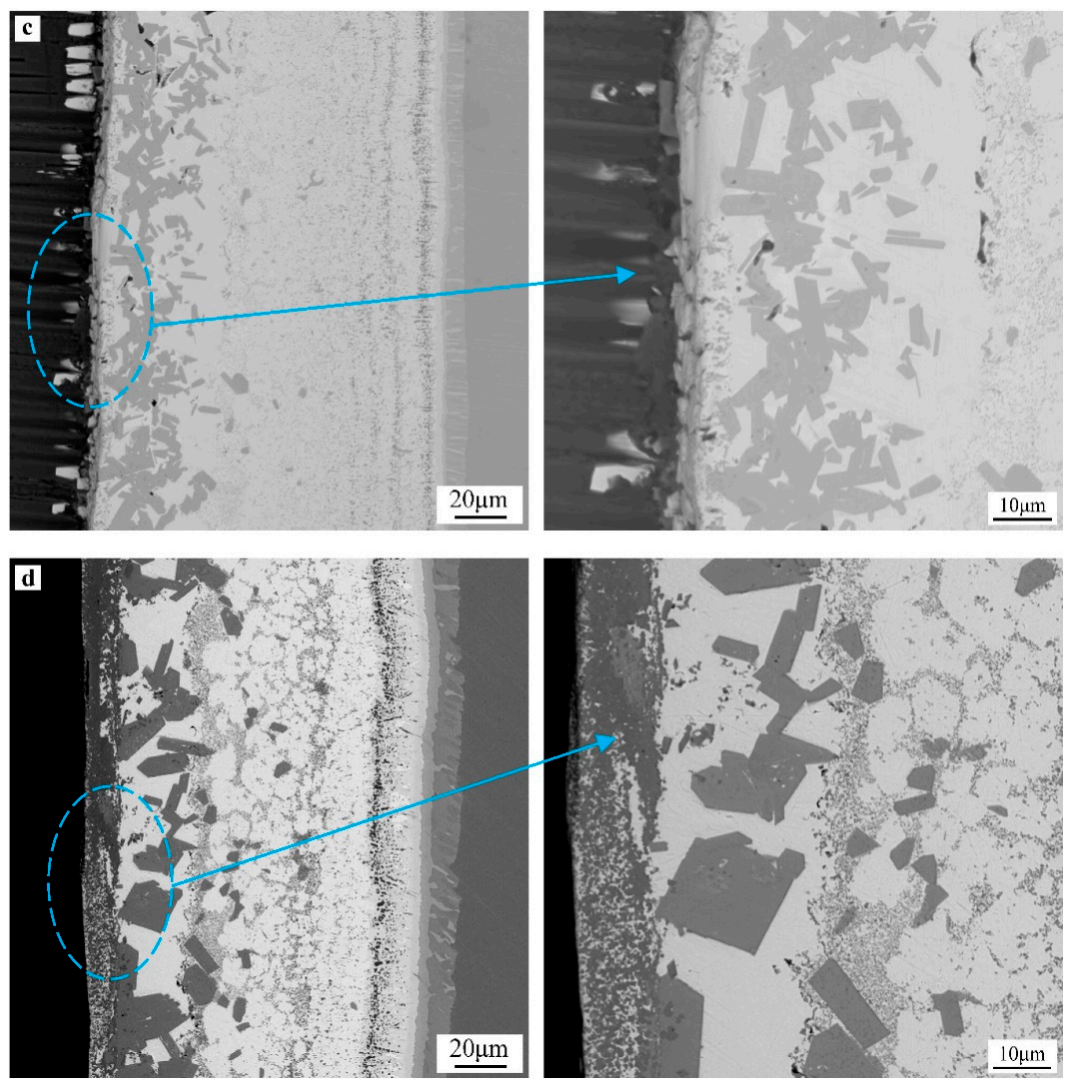
BSE Morphologies of brazed joint under different parameters: (**a**) 1030 °C/20 min, (**b**) 1060 °C/20 min, (**c**) 1090 °C/20 min, (**d**) 1120 °C/20 min.

**Figure 7:**
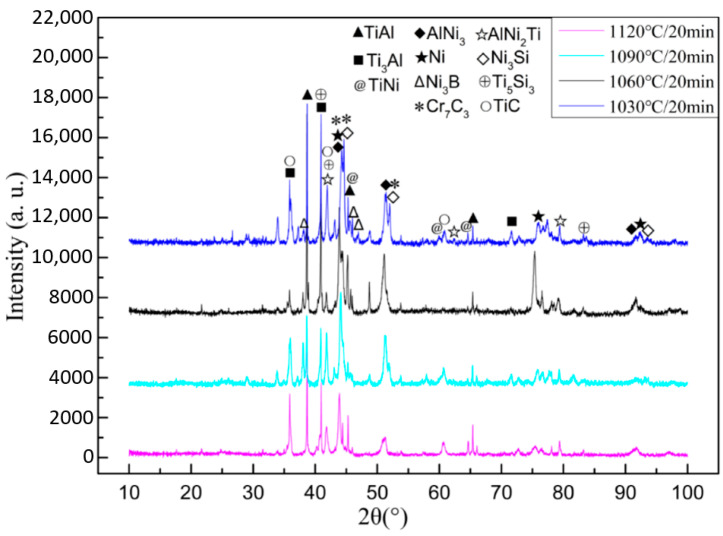
XRD test results of TiAl side under different brazing processes.

Figure 6 shows the cross-section microstructure morphologies of brazed joint at different brazing temperatures. The XRD analysis results of TiAl side interface under different brazing processes is shown in Figure 7. Microstructure of the joint obtained under several brazing temperatures was similar, only the content and distribution state of each phase has some difference.

The microstructures of transition layer I~IV in the interface near TiAl side were the same under different brazing temperature (Figure 6). When brazing temperature was 1120 °C, the microstructure distribution of the joint was obviously different from that at other temperatures. With the increase of brazing temperature, degree of diffusion reaction between filler metal and TiAl alloy increased, resulting in the increase of the thickness of transition layer I. When brazing temperature was 1090 °C, 1120 °C, the thickness of transition layer IV increased significantly. The phase composition of transition layer V~VII was mainly the same as that of BNi-2 alloy under these different brazing temperatures. The difference is that when brazing temperature was 1120 °C, the phase was mainly Ni(s, s), and the content of dispersed compounds was less. Under high brazing temperature, chemical reaction between filler metal and base materials was more easily to carry out, so the compounds’ content at the brazing seam center was reduced.

The morphology and distribution of TiCr_2_ compounds in transition layer VIII were different under different brazing temperature, because of the different diffusion degree of strong carbide forming element Ti, Cr to C/C composites. At 1030 °C, TiCr_2_ particles were small and distributed uniformly. When brazing temperature was 1060 °C, the size of the compound particle increased, its distribution was denser, and the total production increased. At 1090 °C, since more elements were diffused into C/C composites, the size of TiCr_2_ particle was smaller, but the total amount of TiCr_2_ was larger. At 1120 °C, the size of particles increased significantly, and the distribution was relatively sparse.

At the interface of the C/C composite side, active elements react with C element to generate corresponding carbides, that is the prerequisite to realize reliable joining between the filler metal and C/C composite. The category, morphology and distribution of carbides are the main factors affecting the joint performance. As shown in Figure 5a, when brazing temperature was 1030 °C, the amount of (Ti, Cr)C compound near C/C composite was small due to insufficient interface reaction and presented a continuous distribution state. Under 1090 °C (Figure 6c), metal elements diffused more into C/C composite, which changed the original structure of C/C composite at the interface, causing damage and strength decrease of partial composite. When brazing temperature continued to rise to 1120 °C, the content of carbides generated at the interface was large and presented a continuous distribution phenomenon, forming a thick brittle carbide transition layer.

When brazing temperature is lower, the amount of carbide is less, and no stable reaction layer is formed on the brazed joint interface, so the interface bonding is weak and the joint strength is low. When brazing temperature rises to an appropriate temperature, the carbide in the joint presents a fine and diffuse distribution state, and the joint bonding strength increases. When brazing temperature is too high, the thickness of brittle carbide layer increases and the joint performance decreases.

### 3.3. Fracture Morphology Analyses of C/C-BNi2-TiAl Brazed Joint

**Figure 8:**
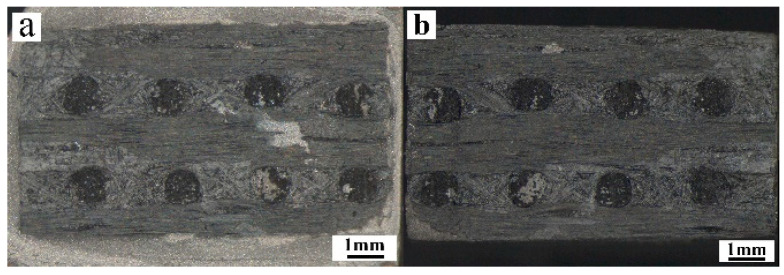
OM macroscopic fracture morphology of brazed joint: (**a**) TiAl side, (**b**) C/C composite side.

**Figure 9:**
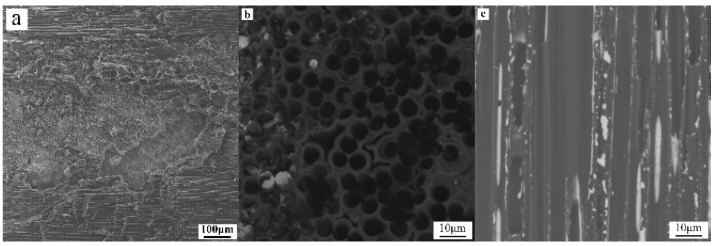
BSE microscopic fracture morphology of brazed joint: (**a**) the overall appearance of the fracture, (**b**) fracture perpendicular to the C fiber, (**c**) fracture parallel to the C fiber.

The macroscopic and microscopic morphology of the fracture is shown in Figure 8 and Figure 9, respectively. It can be seen from the fracture morphology that the fracture showed the brittle fracture of vertical carbon fiber and the tear of paralleled carbon fiber and matrix.

There was brazing filler metal on the fracture surface of the joint (Figure 8), indicating that part of brazing filler metal has penetrated into C/C composite during brazing. Part of C fibers perpendicular to the joint interface was debonded and pulled out from the matrix, leaving holes on the surface of the fracture (Figure 9b). Simultaneously, many reactants were adhered to the surface of C/C composite (Figure 9c), meaning that the brittle reaction layer was also the weak area of the joint, and it was easy to fracture here. The infiltration of filler metal and the diffusion of metal elements changed the original microstructure of C/C composite and decreased the strength of C/C composite at the joint.

## 4. Conclusions

Vacuum brazing of C/C composite and TiAl alloy was realized by using BNi-2 filler metal. The chemical reaction between elements is a necessary condition to realize the metallurgical connection of the joint interface during brazing. New intermetallic compounds such as AlNi_3_, AlNi_2_Ti, TiNi were mainly found on TiAl side. On C/C composite side, there were mainly carbides such as Cr_7_C_3_, Cr_3_C_2_ and TiC. The interface structure of C/C composite and TiAl alloy was complex, and the structure of the cross section is: TiAl alloy → TiAl + AlNi_3_ → AlNi_2_Ti → Ni(s, s) + Ti_3_Al + Ni_3_Si → Ni(s, s) + Ni_3_(Si, B) + CrB → Ni(s, s) + Ni_3_Si + TiCr_2_ → (Ti, Cr)C → C/C composite.

The room temperature shear strength of the joint first increased and then decreased as the brazing temperature increased under the same holding time period. When the brazing temperature was 1060 °C and holding time was 20 min, the average shear strength of the joint reached the highest, which was 11.62 MPa.

The morphology and distribution of carbides on C/C composite side was different under different brazing temperature, which had different influence on the mechanical properties of brazed joint. When the brazing temperature was 1060 °C, the (Cr, Ti)C carbides were fine and dispersed uniformly in brazing seam, and the mechanical property of the joint was the best.

## Figures and Tables

**Table 1:** Basic properties of C/C composite and TiAl alloy [11,12].

Material	Melting Point/°C	Density/g·cm^−3^	Elasticity Modulus/GPa		Coefficient of Thermal Expansion/10^−6^ K^−1^		Thermal Conductivity/W·m^−1^·°C^−1^	
C/C Composite	3600~3800	1.5~2.0	20~100		0~2		60~210	
TiAl alloy	1460	3.87	25 °C	172	100 °C	8.5	100 °C	20.0
					500 °C	11.8	500 °C	22.6
					1000 °C	13.0	1000 °C	22.5

**Table 2:** EDS analyses of typical areas in Figure 3b.

Layer	Area	Chemical Composition (at.%)								Possible Phase
		Ti	Al	V	Ni	Cr	C	Si	Fe	
I	1	55.43	39.61	3.85	-	-	1.11	-	-	TiAl
II	2	34.44	42.36	2.19	19.30	1.72	-	-	-	Ti(Al, Ni) + AlNi_3_
III	3	22.92	29.01	-	46.77	-	-	1.30	-	AlNi_2_Ti
IV	4	43.81	16.20	2.58	33.13	-	-	2.13	-	Ti_3_Al + Ni_3_Si
	5	12.22	19.05	-	67.13	0.28	-	1.32	-	Ni(s, s)
V	6	6.91	4.18	-	32.64	48.47	-	5.59	2.21	Ni_3_Si + CrB
VI	7	34.32	-	2.24	2.67	56.83	3.95	-	-	TiCr_2_
	8	-	10.82	-	73.68	-	-	15.50	-	Ni(s, s) + Ni_3_(Si, Al)
VII	9	3.73	5.73	-	86.32	-	-	-	4.23	Ni(s, s)
	10	0.76	-	-	13.19	74.88	3.92	-	7.26	Ni_3_B+ CrB
VIII	11	-	-	-	76.84	2.14	-	16.77	4.25	Ni(s, s) + Ni_3_Si
	12	33.43	-	-	-	62.45	4.12	-	-	(Ti, Cr)C or TiCr_2_
	13	69.88	-	4.46	-	17.95	7.71	-	-	TiCr_2_ + TiC
IX	14	9.44	-	-	-	75.10	12.70	-	-	Cr-C + TiC

## Data Availability

The data presented in this study are available on request from the corresponding author.
